# Intra-articular administration of autologous adipose-derived stem cells in hip osteoarthritis: Longitudinal treatment trajectories and prognostic factors

**DOI:** 10.1016/j.reth.2025.03.010

**Published:** 2025-03-27

**Authors:** Masaki Hatano, Hisatoshi Ishikura, Tomohiro Terao, Taro Kasai, Ryota Yamagami, Junya Higuchi, Shuichi Yoshida, Yusuke Arino, Ryo Murakami, Masashi Sato, Yuji Maenohara, Yuma Makii, Tokio Matsuzaki, Keita Inoue, Shinsaku Tsuji, Sakae Tanaka, Taku Saito

**Affiliations:** a Department of Orthopaedic Surgery, Faculty of Medicine, The University of Tokyo, Tokyo, Japan; b Ochanomizu Cell Clinic, Tokyo, Japan; c Yakuendai Rehabilitation Hospital, Chiba, Japan; d Avenue Cell Clinic, Tokyo, Japan; e CPC Corporation, Tokyo, Japan

**Keywords:** Adipose-derived stem cell, Prognostic factor, Osteoarthritis severity, Magnetic resonance imaging, Patient-reported outcome, Hip osteoarthritis

## Abstract

**Introduction:**

Evidence regarding the effectiveness of mesenchymal stem cells in improving hip function in patients with hip osteoarthritis (OA) remains inconsistent. Therefore, we aimed to estimate the longitudinal trajectories of treatment effects in individuals with hip OA following intra-articular administration of autologous adipose-derived stem cells (ASCs), both in the overall cohort and the cohort stratified by OA severity, and to explore the prognostic factors of treatment outcomes.

**Methods:**

This retrospective study included adults with hip OA who underwent magnetic resonance imaging (MRI) before treatment between 2020 and 2023. All patients underwent intra-articular ASC administration. The primary outcomes were the total scores on the Japanese Orthopaedic Association Hip Disease Evaluation Questionnaire (JHEQ) at 1, 3, and 6 months after treatment. A linear mixed-effects model was used to estimate the marginally predicted JHEQ total score changes over time for all patients and compare these changes among subgroups categorized according to OA severity. Furthermore, models that included MRI findings were used to explore potential prognostic factors.

**Results:**

We identified 168 hips in 129 patients. In the analysis of all patients, the JHEQ total scores significantly increased at 1, 3, and 6 months after treatment compared with baseline. In the mixed-effects analysis, the predicted change in the JHEQ total score from the baseline to the 6-month follow-up was 10.6 points (95 % confidence interval, 8.3 to 12.8), with an increasing trend over time. The effect modification by OA severity on the JHEQ total score was observed over time after treatment (P = 0.016). Prognostic analysis revealed that patients with moderate hip OA had higher JHEQ total scores after treatment than those with mild hip OA (P = 0.028). Additionally, female patients had higher JHEQ total scores after treatment than male patients (P = 0.025). The presence of ligamentum teres abnormalities was associated with low JHEQ total scores after treatment (P = 0.011).

**Conclusions:**

Intra-articular ASC treatment enhanced patient-reported outcomes over 6 months, with differences observed across hip OA severities. Female sex and moderate hip OA were positive prognostic factors, whereas ligamentum teres abnormalities were negative prognostic factors for patient-reported outcomes after ASC treatment. These findings can be used to inform patients and clinicians about the expected course of treatment and aid clinical decision-making regarding the use of intra-articular ASC treatment for hip OA.

## 1 Introduction

Osteoarthritis (OA) is one of the most prevalent joint disorders that causes chronic pain and reduces quality of life. OA of the knee and hip joints is common among articular joints of the extremities. Conservative therapies, including physical therapy, weight loss, and pharmacological therapy such as oral analgesics or injective treatments, are primarily used to manage joint pain [1]. However, controlling the symptoms of OA or avoiding surgical intervention with these traditional therapies remains challenging. Intra-articular injection of hyaluronic acid is widespread for knee OA; however, it is not available for hip OA in Japan.

Biological therapies using mesenchymal stem cells, stromal vascular fractions, and platelet-rich plasma have attracted attention as novel conservative therapies for managing knee OA. A series of clinical studies have indicated that intra-articular injection of mesenchymal stem cells, particularly autologous adipose-derived stem cells (ASCs), may be effective for joint pain in patients with knee OA. In addition, we recently reported enhanced short-term clinical outcomes after intra-articular injection of ASC in knee OA [2]. In the study, patients with mild-to-moderate knee OA responded better to the intra-articular injection of ASCs than those with severe OA, and we identified several baseline magnetic resonance imaging (MRI) findings associated with clinical outcomes [2].

In contrast to knee OA, there are few reports of biological therapies for hip OA. A few small-scale studies have suggested that intracapsular injections of stromal vascular fraction or mesenchymal stem cells may alleviate pain and improve hip function in patients with hip OA [[3], [4], [5], [6], [7]]. Despite these promising findings, evidence of its effectiveness remains inconsistent, particularly concerning the impact of treatment across different levels of OA severity [4,5]. Moreover, as treatment effects vary across studies [1,[3], [4], [5], [6]], there is a critical need to identify prognostic factors such as patient demographics and structural features observable through imaging, which may help predict patients who are likely to benefit from intra-articular mesenchymal stem cell administration. In this context, baseline MRI findings may reveal structural pathologies associated with hip OA, potentially offering valuable insights into patient selection [[8], [9], [10]].

In this retrospective study, we aimed to:

1. Estimate the longitudinal trajectories of treatment effects following intra-articular administration of autologous ASCs in patients with hip OA, measured at baseline and at 1, 3, and 6 months after treatment, for all patients and subgroups stratified by OA severity.
2. Explore whether patient characteristics and MRI findings are useful in predicting treatment outcomes, thereby identifying the patients most likely to benefit from ASC treatment.

We hypothesized that ASC treatment provides beneficial effects in patients with hip OA, with treatment responses varying according to the patient characteristics and MRI findings.

## 2 Methods

### 2.1 Patient data collection

In this retrospective study, we used data from the Avenue Cell Clinic in Japan, where intra-articular administration of cultured autologous ASCs has been performed since June 2016. Routine assessments are conducted at the clinic to ensure safe therapy administration, including physical examinations, blood tests, MRI scans, and evaluation of the Japanese Orthopaedic Association Hip Disease Evaluation Questionnaire (JHEQ) score.

To evaluate the effectiveness of ASC treatment on hip joint pain and function, the JHEQ scores were collected at 1, 3, and 6 months after treatment. Patients who met the following criteria were excluded from this treatment: hypersensitivity to anesthetics for adipose collection or substances for the manufacturing process, such as egg protein; an allergic reaction to penicillin, streptomycin, or amphotericin B for cell culture; positive pathogenic microbiological tests, including hepatitis B virus, hepatitis C virus, human immunodeficiency virus, and syphilis; age <20 years; pregnancy or lactation; complications with severe trauma; poor understanding of the treatment; and abnormal prothrombin time or activated partial thromboplastin time before treatment. We included adults aged ≥20 years with a primary diagnosis of hip OA who received ASC treatment between April 2020 and December 31, 2023. Patients were excluded if they were lost to follow-up before the 6-month evaluation, did not undergo MRI sequences within 3 months before treatment, or had a history of femoral head osteonecrosis.

### 2.2 Preparation of ASCs

The abdominal subcutaneous adipose tissue was collected under local anesthesia. The collected tissue was immediately transferred to the cell processing center of CPC Corporation. The cells were then isolated from the tissue using an unwoven fabric and cultured in an optimized medium containing 1–4% autologous serum. The cells were cultured at 37 °C with 5 % CO_2_ for 4–5 weeks until a target of approximately 1 × 10^8^ cells was reached [11].

### 2.3 Intra-articular injection of ASCs

Approximately 1 × 10^8^ ASCs were injected into the hip joint space using a 23-gauge needle under ultrasonographic guidance. The patients were not asked to restrict their joint movements or daily activities after ASC treatment.

### 2.4 Outcomes and covariates

The primary outcomes were the JHEQ total scores at 1, 3, and 6 months after ASC treatment. The secondary outcomes were the scores for each JHEQ component (pain, mobility, and mental health) at the same intervals. The JHEQ comprises three subscales: pain (28 points), mobility (28 points), and mental health (28 points), with higher scores indicating better outcomes [12]. The primary outcome, the JHEQ total score at 6 months after ASC treatment, was further assessed in relation to the minimal clinically important difference (MCID) of 9. As the MCID for the JHEQ score has not yet been validated, it was estimated with reference to the MCID of the modified Harris Hip Score in individuals with hip OA [13].

Other factors included age, sex, body mass index, type of occupation (categorized as active, sedentary, or unemployed based on a self-report questionnaire) [14], duration from symptom onset to treatment, OA severity, and MRI factors (including bone marrow lesions, effusion/synovitis, labral abnormalities, ligamentum teres abnormalities, subchondral cysts, and anterior shift sign). Age was subdivided into three categories: 1) ≤ 59 years, 2) 60–69 years, and 3) ≥ 70 years. OA severity was classified as follows: mild (Kellgren-Lawrence grade 1 or 2), moderate (grade 3), and severe (grade 4).

### 2.5 Imaging evaluation

To accurately assess hip joint OA and optimize MRI evaluation, OA severity, and the following six features were analyzed: (i) bone marrow edema, (ii) subchondral cyst, (iii) labral abnormality, (iv) effusion/synovitis, (v) ligamentum teres abnormality, and (vi) anterior shift sign [[8], [9], [10]]. In particular, the bone marrow edema pattern was defined as an ill-defined subchondral lesion hyperintense on fluid-sensitive sequences >1 cm in size. Subchondral cysts were defined as well-defined fluid-signal bone lesions >0.5 cm in size. Labral abnormalities were defined as the presence of abnormal signals, fraying, tears, labral cartilage separation, or macerations. Joint effusion was interpreted as a sign of synovitis and the presence of a fluid signal at the femoral neck region greater than 0.7 cm in thickness. Ligamentum teres abnormalities were defined as signal abnormalities, fraying, or tears. The anterior shift sign was defined as a gap of ≥1 mm between the posterior part of the femoral head and the corresponding acetabular surface. Weighted κ coefficients with 95 % confidence intervals (CIs) were used to evaluate the interobserver reliability of the Kellgren-Lawrence grading system for hip OA on MRI. The κ values indicated the level of agreement as follows: ≤0.20, poor; 0.21–0.40, fair; 0.41–0.60, moderate; 0.61–0.80, good; and 0.81–1.00, excellent [15]. The Kellgren-Lawrence grading system for hip OA demonstrated excellent agreement, with a weighted κ of 0.82 (95 % CI, 0.75–0.88).

### 2.6 Statistical analysis

Data are presented as medians with interquartile ranges or means with standard deviations for continuous variables and numbers and proportions for categorical variables. One-way repeated-measures analysis of variance was used to estimate changes in outcomes over time. When the global F-test indicated statistical significance, pairwise comparisons were performed using Tukey's test. This analysis was also stratified by OA severity.

We used a linear mixed-effects model with random effects, including a random intercept for individuals and a random slope for follow-up time, assuming an unstructured covariance structure to estimate and visualize the marginally predicted JHEQ total score changes over the follow-up period [16]. The model included age, sex, body mass index, treated side, duration from symptom onset to treatment, and OA severity. To test effect modification by OA severity on JHEQ total scores over time after treatment, the P-value for the interaction term between time and OA severity was calculated. We decided *a priori* to investigate the impact of OA severity on the JHEQ total score over time only if the effect modification was significant. Linear mixed-effects models were reanalyzed and stratified by OA severity. Additionally, to identify potential prognostic factors for the primary outcomes, we used models that included MRI findings as additional variables. All P-values were two-sided, with P < 0.05 indicating statistical significance. All statistical analyses were performed using the Stata software (version 17; StataCorp, College Station, Texas, United States) and the Python software (version 3.8; Python Software Foundation).

## 3 Results

### 3.1 Patient characteristics

**Fig. 1:**
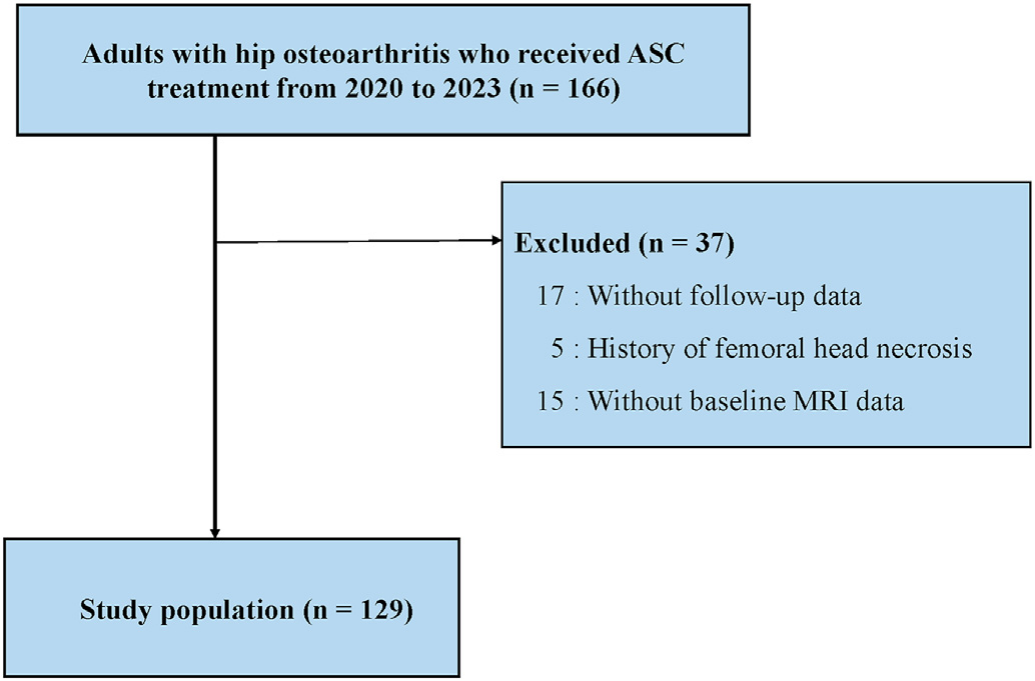
**Flowchart of the current study**. ASC, autologous adipose-derived stem cell; MRI, magnetic resonance imaging.

After applying the inclusion and exclusion criteria, 168 hips from 129 patients were included in the study (Fig. 1). Of these, 31, 39, and 98 hips were classified as mild, moderate, and severe OA, respectively (Table 1). The median age of the patients was 63 years (standard deviation: 9 years), and 26 patients (20 %) were men (Table 1). No significant imbalance in the distribution of OA severity was observed among the three groups (Table 1).

**Table 1:** Patient characteristics according to OA severity: mild, moderate, and severe.

	Total	Mild group	Moderate group	Severe group	P-value
Total patients, n	N = 168	N = 31	N = 39	N = 98	
Treated side (left), n (%)	70 (42 %)	13 (42 %)	14 (36 %)	43 (44 %)	0.690
Mean age, n (%)					0.340
≤59	49 (29 %)	7 (23 %)	15 (38 %)	27 (28 %)	
60-69	84 (50 %)	17 (55 %)	14 (36 %)	53 (54 %)	
≥70	35 (21 %)	7 (23 %)	10 (26 %)	18 (18 %)	
Male sex, n (%)	31 (19 %)	4 (13 %)	12 (31 %)	15 (15 %)	0.074
Mean BMI, kg/m^2^ (SD)	22.3 (3.4)	22.5 (2.8)	22.2 (3.3)	22.2 (3.6)	0.920
Mean duration from symptom onset to treatment, year (SD)	4.4 (6.1)	2.8 (2.6)	6.0 (10.9)	4.3 (3.9)	0.097
Type occupation, n (%)					0.051
Sedentary occupation	58 (35 %)	9 (29 %)	19 (49 %)	30 (31 %)	
Active occupation	39 (23 %)	3 (10 %)	11 (28 %)	25 (26 %)	
No occupation	40 (24 %)	11 (35 %)	4 (10 %)	25 (26 %)	
Unknown	31 (19 %)	8 (26 %)	5 (13 %)	18 (18 %)	

BMI, body mass index; SD, standard deviation; OA, osteoarthritis.

### 3.2 Primary outcomes

**Fig. 2:**
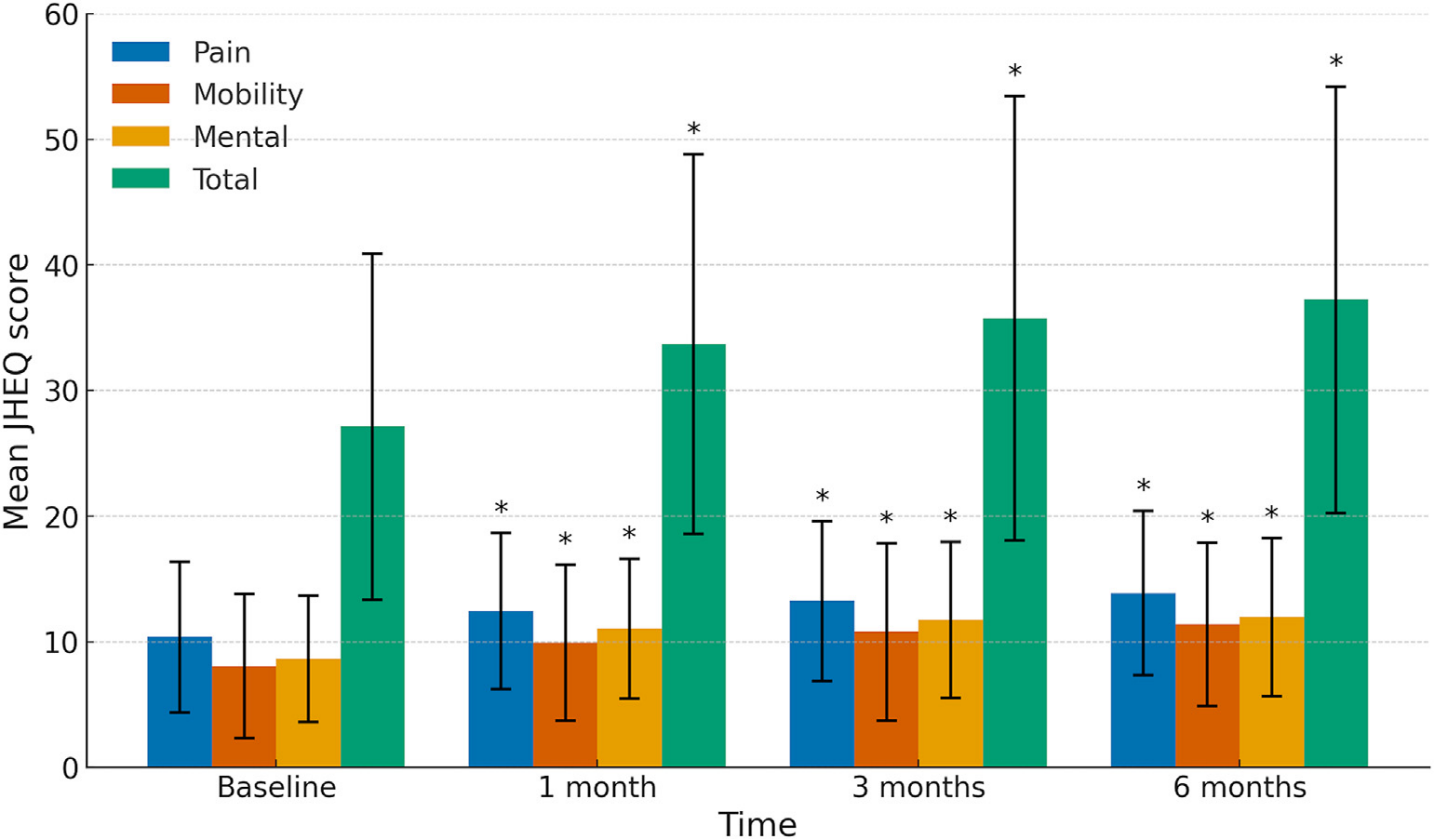
**Chronological changes in JHEQ total and subscale scores.** The JHEQ total and subscale scores at baseline and at 1, 3, and 6 months after treatment are shown. One-way repeated-measures analysis of variance and post hoc pairwise comparisons (Tukey's test) were conducted. ∗P < 0.05, compared with baseline. JHEQ, Japanese Orthopaedic Association Hip-Disease Evaluation Questionnaire.

In the analysis of all patients, the JHEQ total scores significantly increased at 1, 3, and 6 months after treatment compared with baseline (Fig. 2). No statistically significant increase was observed between 1 and 6 months after treatment.

**Fig. 3:**
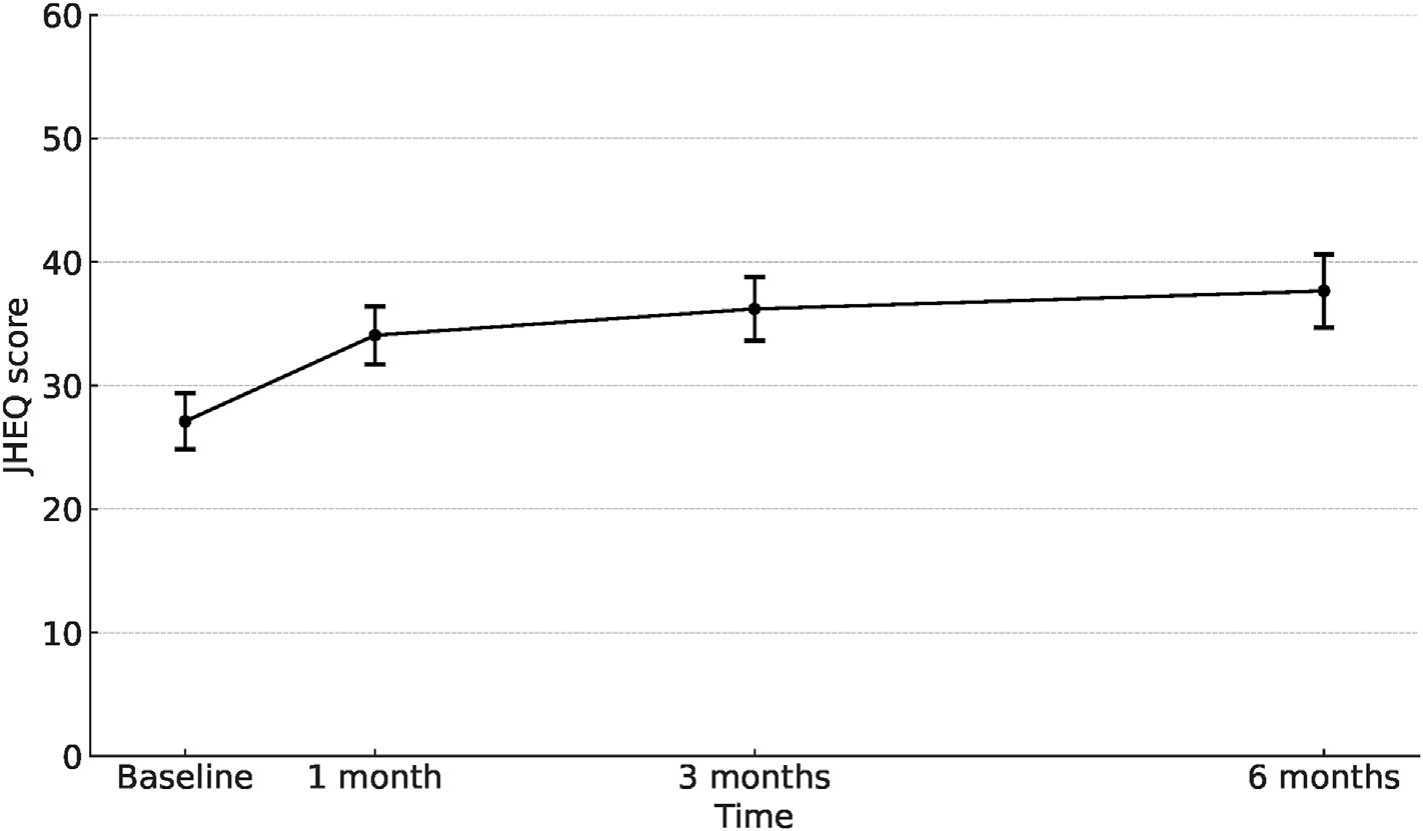
**Marginally predicted changes in JHEQ total score.** The marginally predicted changes in the JHEQ total scores over the follow-up period were evaluated using a linear mixed-effects model with random effects, including a random intercept for individuals and a random slope for the follow-up time. JHEQ, Japanese Orthopaedic Association Hip-Disease Evaluation Questionnaire.

Mixed-effects analysis was used to account for repeated measures for individual patients. This approach is well suited for longitudinal data as it incorporates both fixed effects, such as time, and random effects to account for interpatient variability. In the mixed-effect analysis, the predicted change in the JHEQ total score from the baseline to the 6-month follow-up was 10.6 points (95 % CI, 8.3–12.8), with a tendency toward an increase over time (Fig. 3). This change reached the threshold for the MCID.

### 3.3 Secondary outcomes

Each of the JHEQ subscale scores, namely, pain, mobility, and mental health, demonstrated significant improvement at 1, 3, and 6 months after treatment compared with the baseline (Fig. 2). No statistically significant increase was observed in any of the JHEQ subscale scores between 1 and 6 months after treatment.

### 3.4 Subgroup analysis and prognostic factors

**Fig. 4:**
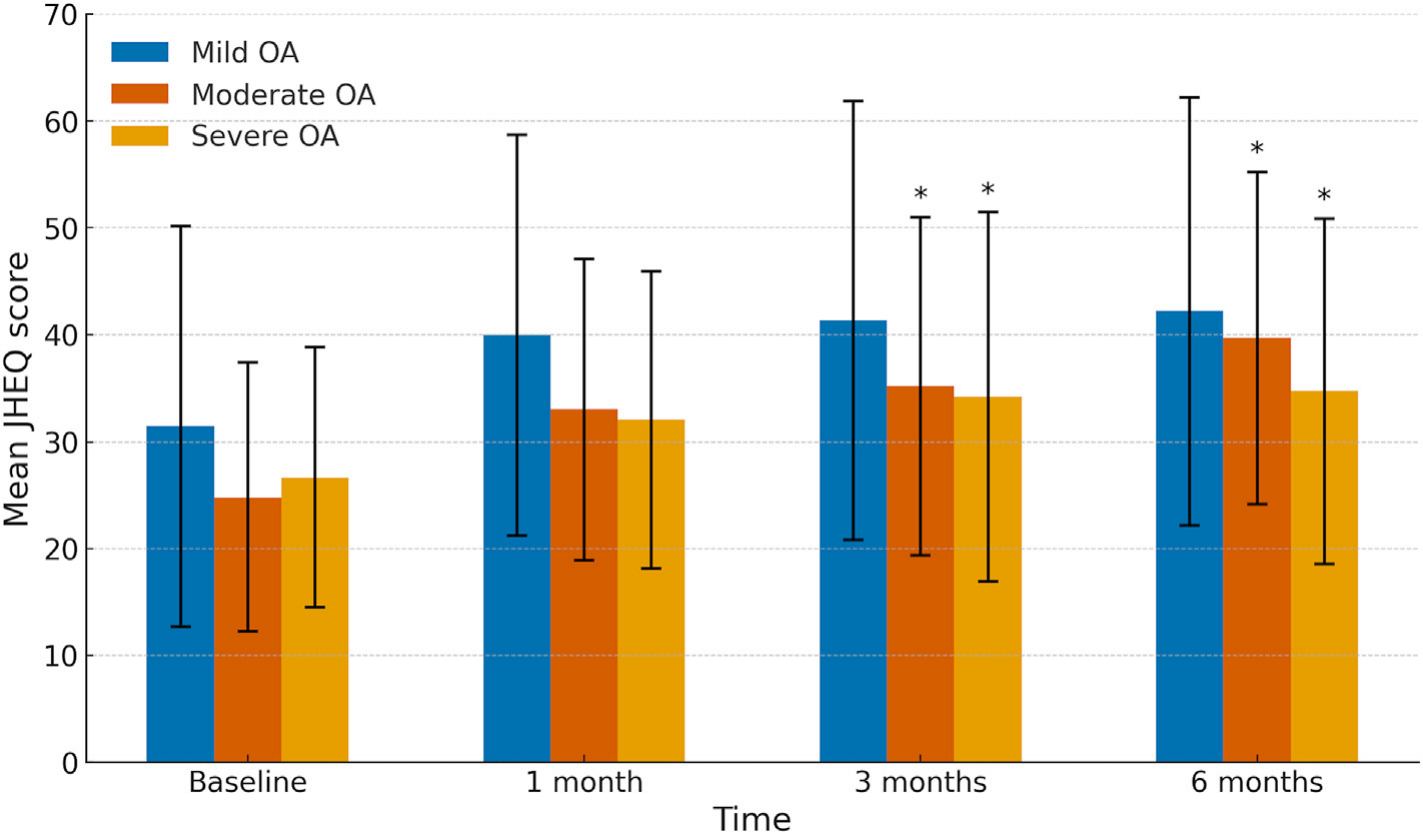
**Chronological changes in JHEQ total scores in patients stratified by OA severity.** The JHEQ total scores for patients with mild, moderate, and severe OA are shown at baseline and 1, 3, and 6 months after treatment. One-way repeated-measures analysis of variance and post hoc pairwise comparisons (Tukey's test) were conducted. ∗P < 0.05, compared with baseline. JHEQ, Japanese Orthopaedic Association Hip-Disease Evaluation Questionnaire; OA, osteoarthritis.

A subgroup analysis was performed based on OA severity (mild, moderate, and severe) to evaluate the differences in JHEQ total score changes over time. In the mild group, the JHEQ total scores after treatment showed no significant increase compared with the baseline (Fig. 4). In contrast, in the moderate and severe groups, the JHEQ total scores at 3 and 6 months after treatment were significantly higher than those at the baseline (Fig. 4).

**Fig. 5:**
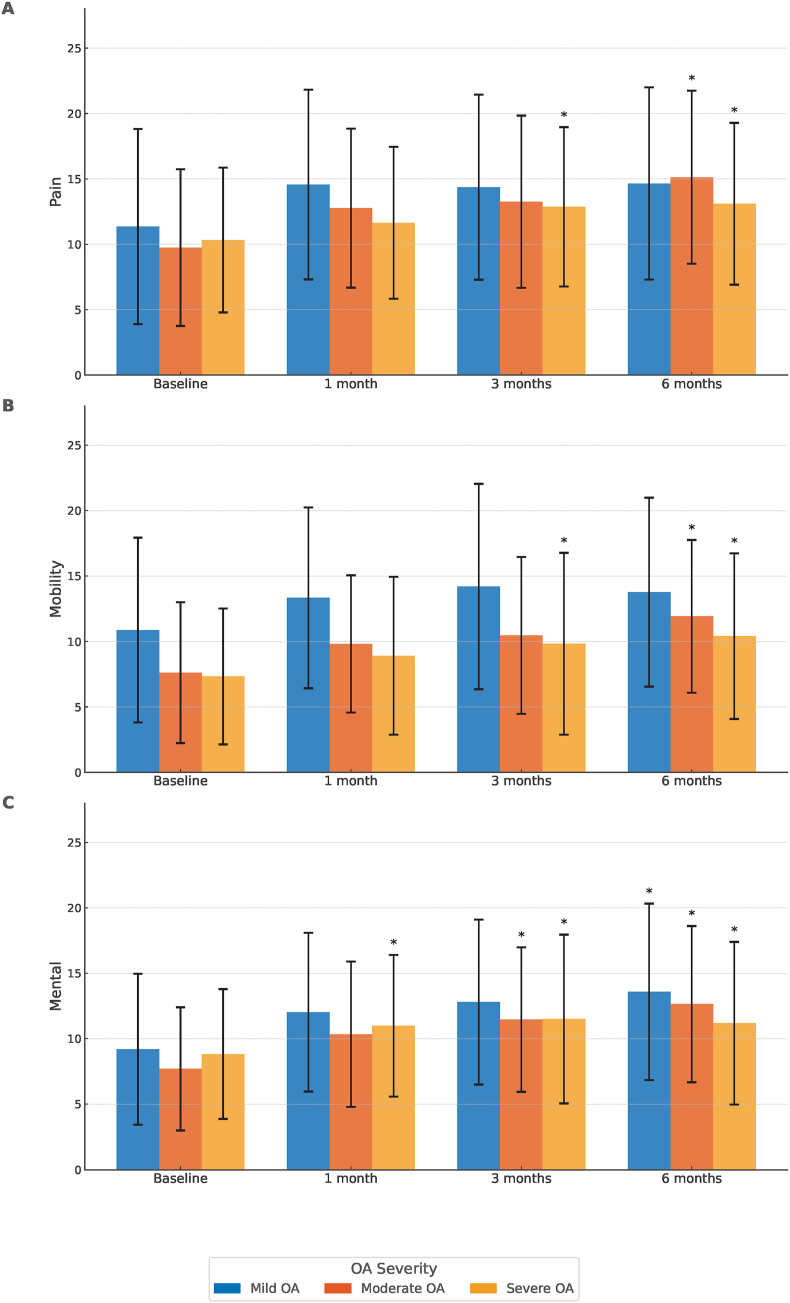
**Chronological changes in JHEQ subscale scores in patients stratified by OA severity**. The JHEQ subscales for pain (a), mobility (b), and mental health (c) are presented for patients with mild, moderate, and severe OA at baseline and 1, 3, and 6 months after treatment. One-way repeated-measures analysis of variance and post hoc pairwise comparisons (Tukey's test) were conducted. ∗P < 0.05, compared with baseline. JHEQ, Japanese Orthopaedic Association Hip-Disease Evaluation Questionnaire; OA, osteoarthritis.

A subgroup analysis based on OA severity was conducted to evaluate changes in the JHEQ subscale scores (pain, mobility, and mental health) over time. In the mild group, only the mental health component showed a significant increase at 6 months after treatment compared with the baseline (Fig. 5a–c). In the moderate group, all components showed significant increases at 6 months after treatment compared with the baseline (Fig. 5a–c). In the severe group, significant increases were observed in all components at both 3 and 6 months after treatment compared with the baseline (Fig. 5a–c). No significant differences in any subscale scores were observed across any OA severity level between 1 and 6 months after treatment.

**Fig. 6:**
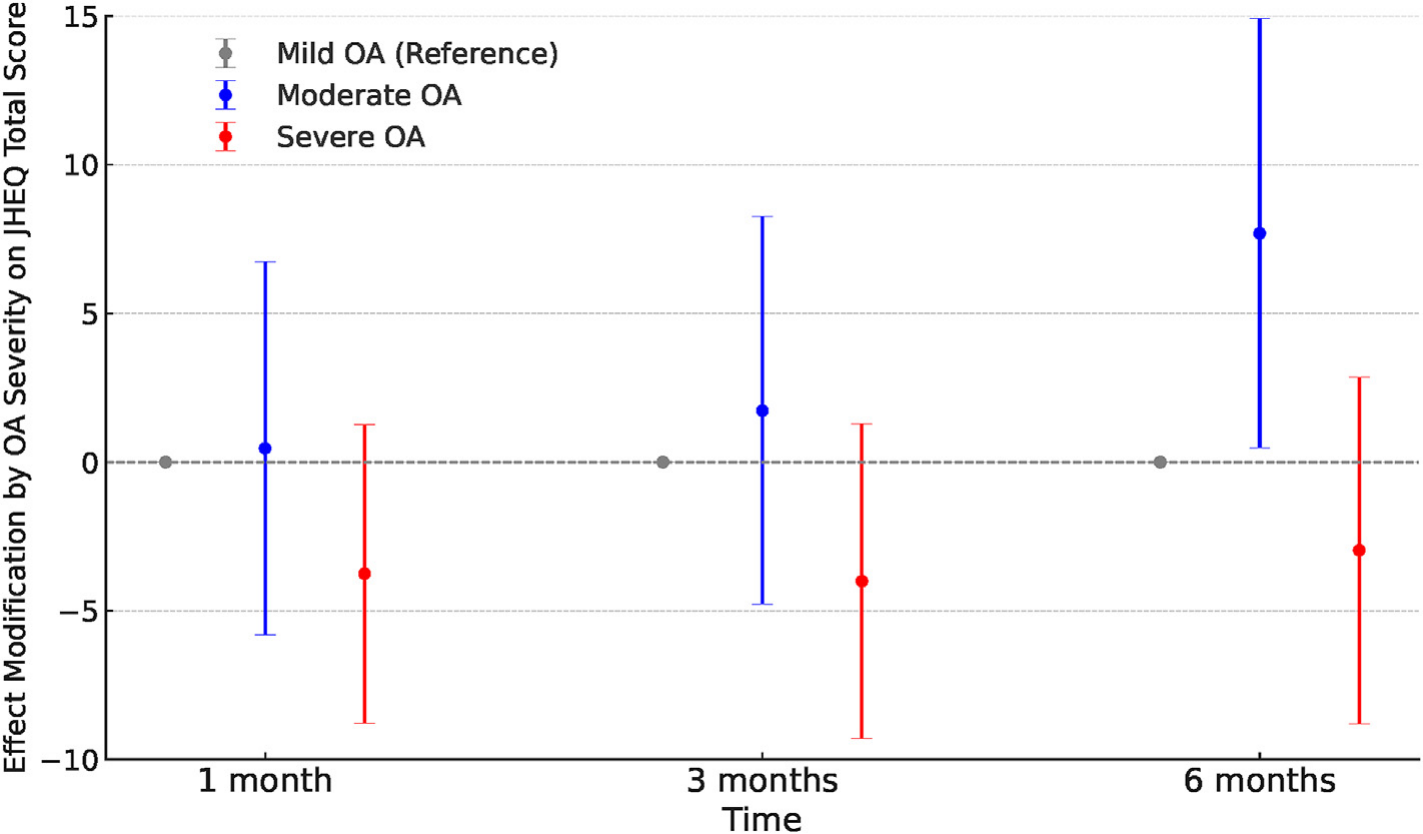
**Effect modification by OA severity on JHEQ total scores over time**. Effect modification, represented as changes in the JHEQ total scores over time, with mild OA as the reference group, was calculated from the interaction terms between time and OA severity using a linear mixed-effects model. The P-value for the modification was 0.016. JHEQ, Japanese Orthopaedic Association Hip-Disease Evaluation Questionnaire; OA, osteoarthritis.

In the mixed-effects analysis, the effect modification by OA severity on the JHEQ total score was detected over time after treatment (P = 0.016) (Fig. 6). The estimated change in the JHEQ total score was greater in patients with moderate OA than in those with mild OA at 6 months after treatment. Although not statistically significant, the estimated change in the JHEQ total score tended to be lower in patients with severe OA than in those with mild OA across months 1–6 (Fig. 6).

**Fig. 7:**
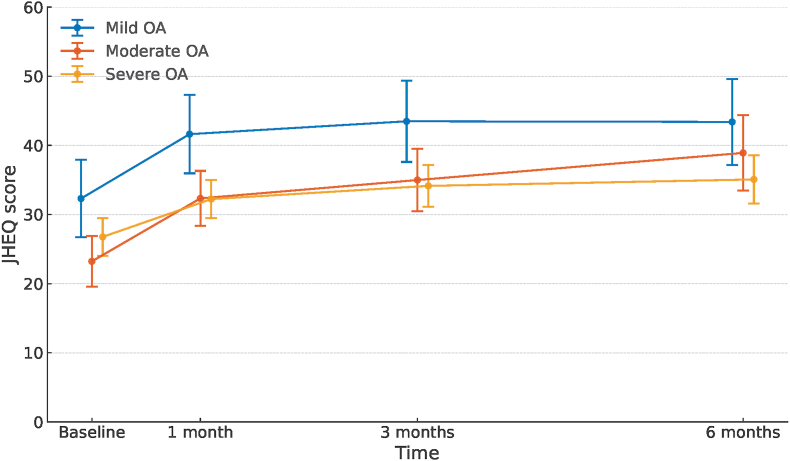
**Marginally predicted changes in JHEQ total score in patients stratified by OA severity**. The marginally predicted changes in the JHEQ total scores for patients with mild, moderate, and severe OA over the follow-up period were evaluated using a linear mixed-effects model with random effects, including a random intercept for individuals and a random slope for the follow-up time. JHEQ, Japanese Orthopaedic Association Hip-Disease Evaluation Questionnaire; OA, osteoarthritis.

In the mixed-effects analysis stratified by OA severity, the predicted changes in the JHEQ total score from the baseline to 6 months were 11.1 points (95 % CI, 7.3–14.9), 15.7 points (95 % CI, 11.4–20.0), and 8.3 points (95 % CI, 5.3–11.3) in the mild, moderate, and severe groups, respectively (Fig. 7). The moderate group demonstrated a trend of a monotonic increase in the score over time, whereas the mild and severe groups showed a trend toward a plateau at 3 months after treatment (Fig. 7). The changes in the mild and moderate groups reached the threshold for the MCID; however, the change in the severe group did not reach this threshold.

**Fig. 8:**
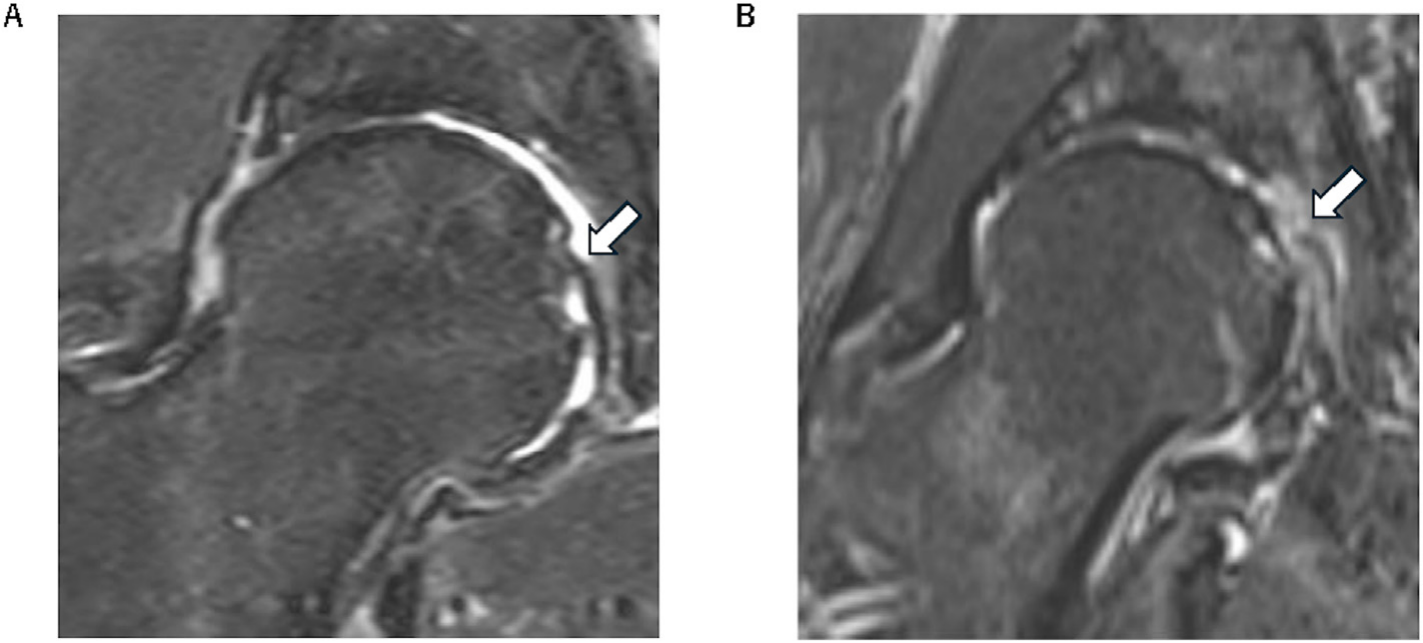
**Ligamentum teres on coronal T2-weighted fat-suppressed images**. (A) Representative image of the ligamentum teres (arrow) without evidence of tear. (B) Representative image of torn ligamentum teres. The arrow indicates discontinuity of the ligament.

In the prognostic analysis, patients with moderate hip OA had a 7.9-point higher JHEQ total score at 6 months after treatment than those with mild hip OA (P = 0.028) (Table 2). In contrast, patients with severe hip OA showed no significant difference in the JHEQ total score at 6 months after treatment compared with those with mild hip OA (P = 0.706) (Table 2). Women had a 6.8-point higher JHEQ total score at 6 months after treatment than men (P = 0.025) (Table 2). Patients with ligamentum teres abnormalities had a 6.4-point lower JHEQ total score than those without ligamentum teres abnormalities (P = 0.011) (Table 2, Fig. 8).

**Table 2:** Prognostic mixed-effect model of baseline patient demographic and magnetic resonance imaging findings for improvement in JHEQ score.

Fixed effects parameter	β (6 months after treatment)	95 % CI	P*-*value
OA severity			
Mild × month	Reference		
Moderate × month	7.9	0.8, 14.9	0.028
Severe × month	−1.2	−7.4, 5.0	0.706
Age category			
≤59 years × month	Reference		
60–69 years × month	1.1	−4.5, 6.6	0.705
≥70 years × month	1.5	−5.1, 8.0	0.658
Sex			
Male × month	Reference		
Female × month	6.8	0.8, 12.8	0.025
Type of occupation			
No occupation × month	Reference		
Sedentary occupation × month	−1.8	−8.0, 4.5	0.581
Active occupation × month	−1.9	−8.8, 5.0	0.582
MRI findings			
Labral abnormality × month	1.5	−3.6, 6.6	0.557
Effusion/synovitis × month	0.3	−4.1, 4.6	0.897
Subchondral cyst × month	−2.5	−7.2, 2.2	0.304
Bone marrow edema × month	1.9	−3.0, 6.8	0.445
Ligamentum teres abnormality × month	−6.4	−11.3, −1.4	0.011
Anterior shift sign × month	0.0	−5.7, 5.6	0.986

JHEQ, Japanese Orthopaedic Association Hip Disease Evaluation Questionnaire; OA, osteoarthritis; MRI, magnetic resonance imaging; CI, confidence interval.

## 4 Discussion

Our findings demonstrated that intra-articular ASC treatment for hip OA enhanced the JHEQ total scores at 1, 3, and 6 months compared with baseline, showing a monotonic trend of score increase over time. Additionally, we identified the effect modification by OA severity on the JHEQ total scores over time after treatment. The changes in the JHEQ total scores reached the threshold for the MCID in the mild and moderate groups indicating that they were likely clinically important. However, the changes in the severe group did not reach this threshold. The analysis of the potential prognostic factors for treatment response revealed that female sex and moderate hip OA were positive prognostic factors, whereas ligamentum teres abnormalities were negative prognostic factors for patient-reported outcomes after ASC treatment. These findings enhance our understanding of intra-articular ASC treatment for hip OA and highlight the patient factors that may influence patient-reported outcomes.

Regenerative therapy using mesenchymal stem cells has the potential to improve the symptoms of patients with hip OA [17]. Six of the seven prior clinical studies reported improvements in patient-reported outcomes and the absence of major complications following mesenchymal stem cell injections [5,17]. Despite these promising findings, substantial variability has been observed in patient-reported outcomes after treatment across studies [[3], [4], [5], [6], [7],^17^]. This variability is likely due to inadequate adjustment for differences in patient demographics and baseline OA severity as well as the small sample sizes in these studies (n ≤ 42), limiting the ability to precisely estimate the overall marginal treatment effect and to differentiate treatment effects based on factors such as hip OA severity. To address these limitations, we included 168 hips from 129 patients and used linear mixed-effects models to provide a robust assessment of the effectiveness of treatment using longitudinal data, including patient demographics and hip OA severity. Our results suggest that intra-articular ASC treatment for hip OA may result in continued improvement in JHEQ total scores over a 6-month period, with differences observed across hip OA severities.

Our findings suggest that the severity of hip OA modifies patient-reported outcomes over time after ASC treatment. Patients with severe knee OA have been shown to respond less effectively to intra-articular ASC treatment than those with mild-to-moderate knee OA [2]. These results align with our findings that patients with severe hip OA showed the least overall improvement in JHEQ scores and recorded the lowest JHEQ scores among the three OA severity groups 6 months after treatment. Patients with moderate hip OA showed the most significant improvement in the JHEQ total scores from baseline to 6 months after treatment, reflecting the largest change from baseline among the three groups. However, their absolute scores at 6 months were lower than those of patients with mild OA. In contrast, patients with mild OA achieved the highest absolute scores at 6 months after treatment; however, the overall improvement in the JHEQ total scores after treatment was moderate. This trend suggests that patients with moderate OA who initially present with more severe symptoms are likely to experience greater clinical benefits than those with mild OA. Conversely, the moderate improvement observed in patients with mild OA may reflect limited potential for further improvement. As the JHEQ total score is calculated as the sum of scores from the pain, mobility, and mental health domains, patients with mild OA who have relatively high baseline scores in individual domains may have minimal scope for further improvement, which could constrain their overall post-treatment gains. These findings warrant further studies to explore the mechanisms underlying these trends.

To the best of our knowledge, this is the first study to assess whether patient demographics and MRI findings can predict changes in JHEQ total scores over a 6-month period in patients with hip OA after intra-articular ASC treatment. Our prognostic analysis showed that moderate hip OA and female sex were associated with high JHEQ total scores after treatment, whereas ligamentum teres abnormalities were associated with low JHEQ total scores after treatment. Ligamentum teres is recognized as a stabilizing structure of the hip joint. Its abnormalities may lead to hip joint instability [18,19], which could, in turn, affect patient-reported outcomes after treatment.

This study has several limitations. First, although adjustments were made for multiple variables within the dataset, the analysis did not fully account for potential confounding factors such as patient lifestyle, adherence to treatment protocols, and understanding of the intervention. These factors could have introduced bias into the observed results, potentially compromising their validity. Future research should incorporate strategies to measure and control these factors to enhance the robustness of findings. Second, the follow-up period was relatively short. Although ASC injection for hip OA improved patient-reported outcomes within this timeframe, whether these effects are sustained over a longer period remains unclear. Longer follow-up is crucial to capture the natural progression of OA and assess whether early improvements translate into long-term clinical benefits. Without this data, it is difficult to determine whether the observed changes reflect temporary symptom relief or a change in disease progression. Additionally, a longer observation period is necessary to ensure the detection of delayed adverse events that might not become apparent within the first six months. Delayed adverse events can affect the risk-benefit assessment of the treatment. Therefore, extended follow-up would provide a more accurate evaluation of both efficacy and safety over time. Consequently, further studies with longer follow-up periods and larger sample sizes are required to evaluate sustained improvements, disease modification potential, and late-onset complications. Third, the study did not include a control or standard treatment group because it was not designed as a clinical trial but rather as an analysis of real-world treatment outcomes in a single facility. Consequently, the absence of a placebo or standard treatment group limits the ability to determine the true efficacy of ASC treatment. The inclusion of a control group would provide a more robust basis for comparing the effectiveness of the intervention and accounting for potential placebo effects. Moreover, the lack of randomized controlled trials in this area underscores the need for further research under controlled conditions, particularly given that key treatment parameters—such as the optimal number of stem cells and the ideal dosing schedule—remain unclear. Future research should begin with small-scale feasibility studies to refine treatment protocols, followed by larger, pragmatic controlled trials that reflect routine clinical practice. This approach would provide a more robust basis for comparing intervention effectiveness and facilitate the integration of ASC therapy into standard care for hip OA. Finally, post-treatment imaging assessments were not performed in this study. This omission limits our ability to explore structural changes in the hip joint, such as cartilage repair, synovial inflammation, or bone remodeling, which could provide valuable insights into the mechanism of action of intra-articular ASC treatment. Understanding these structural changes is crucial to determining whether the observed clinical improvements correspond to actual disease modification. To address this limitation, future studies should incorporate serial imaging—with a baseline scan and subsequent follow-up scans at defined intervals (e.g., 6 and 12 months). Advanced imaging techniques, such as T2 mapping or delayed gadolinium-enhanced MRI of cartilage, can detect subtle changes in cartilage, while ultrasound can further assess joint effusion, synovial thickening, and synovitis [20]. Incorporating these imaging modalities will help clarify whether ASC treatment leads to genuine disease modification.

## 5 Conclusions

In conclusion, our results revealed that among patients with hip OA, that intra-articular ASC treatment resulted in clinical improvement over 6 months, with differences observed across OA severities. Female sex and moderate hip OA were positive prognostic factors, whereas ligamentum teres abnormalities were negative prognostic factors for patient-reported outcomes after ASC treatment. These findings can be used to inform patients and clinicians about the expected course of treatment and aid clinical decision-making regarding the use of intra-articular ASC treatment for hip OA.

## Informed consent

Written informed consent was obtained from all the patients. All the data were handled in accordance with the Declaration of Helsinki.

## Authors’ contributions

MH conceived and designed the study, collected and analyzed the data, and drafted the manuscript. HI, TT, TK, RY, JH, SY, YA, RM, MS, Yuj M, Yum M, TM, and TS conducted the ASC therapy and collected the data. KI and ShT managed ASC preparation. SaT supervised clinical studies. All authors have approved the manuscript. TS provided advice on the study design, interpreted the data, and revised the manuscript.

## Ethical approval

Ethical approval for the study protocol was obtained from the Institutional Review Board (approval numbers: 2018100NI and 2023392NI).

## Funding

This study received no funding.

## Declaration of competing interest

MH, HI, TT, TK, RY, JH, SY, YA, RM, MS, Yuj M, Yum M, and TS were affiliated with Ochanomizu Cell Clinic, where patients were recruited, the ASC treatment was administered, and follow-up was conducted. Because this study was not a clinical trial, it did not receive any funding, and the authors have no financial or nonfinancial interest in the subject matter.
